# Supplementary material for: Complexome profiling of the Chlamydomonas psb28 mutant reveals TEF5 as an early PSII assembly factor
Source: Plant Cell. 2025 Mar 17;37(6):koaf055. doi: 10.1093/plcell/koaf055 (PMC12164590; doi:10.1093/plcell/koaf055)
Supplement: koaf055_Supplementary_Data [file koaf055_supplementary_data.zip › psb28_interactive.zip!/plots/intensity/qualityPlots/peptides/_Cre07.g331850.t1.1.html]

### Cre07.g331850.t1.1

Trivial name:   
SeqCoverage [median]: 8.60  
Mass: 46.33 kDa  
  
 

| Sequence | MissedCleavage | Mass [Da] | wtIntensity | muIntensity | Score |
| --- | --- | --- | --- | --- | --- |
| LTYALTLLR | 0 | 1062.64 | 259.94 | 0.00 | 69.42 |
| VLLERPPPPTGK | 1 | 1302.77 | 0.00 | 772.25 | 38.63 |
| VPPEPAAAAVTDVGLVLVR | 0 | 1873.07 | 634.69 | 0.00 | 48.88 |
|  |  |  | 894.6 | 772.3 |  |
